# S.M.A.R.T. Flex vascular stent system in femoropopliteal arteries: 18-month result of a real-world registry

**DOI:** 10.1186/s40001-025-02904-w

**Published:** 2025-07-22

**Authors:** Meng Ye, XiangJiang Guo, Qihong Ni, Zhenyu Shi, Lianrui Guo, Xixiang Gao, Zibo Feng, Zhu Tong, Xiang Wang

**Affiliations:** 1 Department of Vascular Surgery, Renji Hospital, School of Medicine, Shanghai Jiaotong University, Shanghai, China; 2 Department of Vascular Surgery, Zhongshan Hospital, Fudan University, Shanghai, China; 3 Department of Vascular Surgery, Xuanwu Hospital Capital Medical University, Beijing, China; 4 Department of Vascular Surgery, Liyuan Hospital Affiliated Tongji Medical College of Huazhong University of Science and Technology, Wuhan, China; 5 Department of Vascular Surgery, Shanghai East Hospital, School of Medicine, Tongji University, Shanghai, 200120 China

**Keywords:** S.M.A.R.T. Flex, Femoropopliteal, Peripheral artery disease, Chronic limb-threatening ischemia, Clinically driven target lesion revascularization

## Abstract

**Objective:**

To evaluate the efficacy and safety of the S.M.A.R.T. Flex stents for treating femoropopliteal (FP) occlusive lesions, including complex Trans-Atlantic Inter-Society Consensus (TASC) II C/D and severely calcified lesions.

**Methods:**

This retrospective study utilized the TALENT registry database (a prospective study), enrolling patients who underwent S.M.A.R.T. Flex implantation from January 2021 to July 2022. The primary effectiveness endpoint was the rate of freedom from clinically driven target lesion revascularization (CD-TLR) at 18 months, and the primary safety endpoint was the 18-month rate of freedom from major adverse events (all-cause death, above-the-ankle target limb amputation, or CD-TLR).

**Results:**

A total of 122 patients with 124 limbs were included, with an average follow-up of 617 days. The average lesion length was 24.9 ± 20.4 cm, and 73.8 and 54.1% of the lesions had chronic total occlusions and chronic limb-threatening ischemia, respectively. A total of 127 stents were placed in 107 limbs, with 1 stent placed in 88 limbs (82.2%), 2 stents placed in 18 limbs (16.8%), and 3 stents implanted in 1 limb (0.9%). In this study, 50.5% of the limbs were treated with DCBs, and 83.3% of the TASC C/D lesions were treated primarily with DCBs. The 12- month rate of primary patency was 73.1%. The 18-month rate of freedom from CD-TLR was 93.9% (95% CI: 89.2–98.8%), and the major adverse event-free rate was 75.7% (95% CI: 68.0–84.3%). A total of 92.5% of patients showed primary sustained clinical improvement, and the Vascular Quality of Life Questionnaire scores were significantly improved at 18 months compared with baseline.

**Conclusions:**

The S.M.A.R.T. Flex stents demonstrate promising efficacy and safety as a treatment option for long-segment complex femoropopliteal artery lesions.

## Introduction

The femoropopliteal (FP) artery is commonly involved in peripheral artery disease (PAD). Due to the long length of the lesion and the mobile nature of this arterial segment, it tends to have a high restenosis rate after endovascular therapy. Historically, surgical bypass has been recommended as the gold-standard treatment for complex TransAtlantic Inter-Society Consensus (TASC) II, C and D lesions of FP occlusive lesions [1]. However, not all patients with challenging comorbidities are eligible for bypass surgery, and the procedure is also limited by the patency of the distal runoff, graft type, and the quality of the venous grafts.

Compared with plain old balloon angioplasty (POBA), self-expanding nitinol stents have been shown to significantly improve the patency of endovascular treatment of PAD [2]. However, conventional nitinol stents are susceptible to fatigue and fracture in the FP segment due to various biomechanical forces, including flexion, elongation, compression, torsion, shortening, and kinking. Additionally, the restenosis rates after stenting in the long FP lesion were high, ranging from 55 to 65% at 1 year [3–6]. The S.M.A.R.T. Flex stents (Cordis US Corp.) were specially designed to provide good conformability and resistance to fracture. Nonetheless, there is limited experience with these stents, mostly limited to short or intermediate lesions in patients with Rutherford stages of less than 4. The device’s performance in more challenging lesions, such as TASC C/D lesions or in the presence of severely calcified vasculature, has not been published.

We accessed the TALENT registry to evaluate the safety and effectiveness of S.M.A.R.T. Flex for treating patients with FP occlusive lesions, even those with complex TASC C/D and severely calcified lesions. As the TALENT study received prior Institutional Review Board (IRB) approval at all participating centers, this study was exempt from additional IRB review.

## Methods

### Patients

The TALENT registry is an ongoing study that has been conducted at 11 centers in China since January 2021. These centers include the following: (1) The Hospital of Chengdu University of Traditional Chinese Medicine; (2) The First Affiliated Hospital of Medicine College of Zhejiang University; (3) Liyuan Hospital Affiliated to Tongji Medical College of Huazhong University of Science and Technology; (4) Xuanwu Hospital Affiliated to the Capital Medical University; (5) Qingdao Haici Hospital Affiliated to Qingdao University; (6) Hangzhou First People’s Hospital Affiliated to Zhejiang University; (7) The Second Affiliated Hospital of Soochow University; (8) Zhongshan Hospital of Fudan University; (9) Renji Hospital of Shanghai Jiaotong University; (10) Shanghai East Hospital Affiliated to Tongji University; (11) Shanghai General Hospital Affiliated to shanghai Jiaotong University. All patients signed their written informed consent before the study initiation. Briefly, TALENT is a prospective, multicenter patient registry focused on evaluating how below-the-knee runoff influences patency rates following EVT for FPOD in real-world settings. The details of this study have been previously published [7]. Baseline features, demographics, procedural data, complications during the patient’s hospital stay, clinical outcomes, and any adverse events or major adverse events during follow-up are recorded in the VASCBASE database. The TALENT registry study protocol was approved by the Medical Ethics Committee of the Affiliated Hospital of Chengdu University of Traditional Chinese Medicine (No. 2020KL-078) and was conducted in accordance with the Helsinki Declaration of 1975 as revised in 2013. The study protocol has been registered at Clinicaltrials.gov (Identifier NCT04675632). And in our study, to protect patient information, we de-identified all patient details. The reporting of this study conforms to the STROBE guidelines [8]. This study has been checked with our review board and received its exemption.

The inclusion criteria of the TALENT registry study are as follows:

1. Presence of severe stenosis (≥70%) or occlusive disease in the femoropopliteal artery.
2. Subjects must comprehend the study’s purpose and agree to participate in the clinical study and cooperate with postoperative follow-up.
3. Informed consent must be provided by the subjects.

Exclusion criteria

Any subject meeting any of the following criteria will be excluded from this study:

1. Patients presenting with acute arterial thrombosis or embolism.
2. Serum creatinine higher than 2 mg/dL.
3. Rutherford category of 5 with an infection score of 2 or 3 as determined by the Wound, Ischemia, and Foot Infection classification.
4. Patients with prior femoral-popliteal bypass surgery.
5. Patients with documented hypersensitivity to contrast media, paclitaxel, or antithrombotic agents.
6. Patients with coagulation abnormalities.
7. Pregnant or lactating women.
8. Patients with unstable angina, myocardial infarction, transient ischemic attack or stroke within the past 3 months.
9. Patients with concomitant severe diseases, such as liver failure.
10. Patients with a life expectancy < 24 months.
11. Patients concurrently enrolled in other clinical trials.
12. Non-compliant patients (such as persistent postoperative smoking).

Electronic data were consecutively extracted from this registry’s medical records database for patients who underwent S.M.A.R.T. Flex implantation between January 2021 and July 2022. The extracted data included clinical and procedural records, patient and lesion characteristics, procedural information, outcomes, and follow-up data, all of which were analyzed retrospectively.

### Treatment and medical therapy

All procedures were carried out under local anesthesia. Depending on the location of the target lesion, a contralateral or ipsilateral femoral approach was taken. In general, after the lesions were crossed with a 0.035 or 0.018-inch guidewire, standard balloon angioplasty (POBA) was performed for vessel preparation.

The Principal Investigator (PI) determined whether to perform stent implantation or adjunctive drug-coated balloon (DCB) therapy based on individualized clinical assessment. Following lesion pre-dilation adequately using balloons sized according to the distal reference vessel diameter and anatomical location, a S.M.A.R.T. Flex stent (the size was matched or up to 20% oversizing relative to the balloon diameter used in lesion preparation) should be deployed, ensuring complete lesion coverage. When multiple stents are necessitated, ≥2 cm longitudinal overlap between consecutive stents was mandatory.

The DCB diameter was matched or up to 0.5 mm oversizing to the pre-dilation balloon. DCB inflation duration was maintained for 3 min. Bailout stenting with S.M.A.R.T. Flex stent was required for flow-limiting dissections or residual stenosis >30%.

Dual antiplatelet therapy (DAPT) with daily aspirin 100 mg and clopidogrel 75 mg was administered for ≥6 months, followed by lifelong single antiplatelet therapy with either aspirin or clopidogrel.

### Follow-up

All patients underwent a clinical examination before being discharged. This examination involved evaluating their clinical symptoms based on changes in the Rutherford class and ankle brachial index (ABI). Following their discharge, routine telephonic follow-ups were conducted at 1, 3, 6, 12, 18 and 24 months, and duplex ultrasound follow-up was recommended at 12 and 24 months. The standardized telephonic follow-up protocol included assessing the patients’ medical history and symptoms related to PAD, changes in their quality of life, documenting any adverse events and pharmacotherapy. If a patient experienced clinical deterioration, they were typically referred to the center for re-intervention. Information on subsequent revascularization procedures of the target vessel, as well as other territories, was captured.

### Study endpoints

Technical success was defined as successful vascular access and completion of the endovascular procedure and immediate morphological success with less than 30% residual diameter reduction of the treated lesion on completion of angioplasty. Restenosis was defined as a >2.4 times increase in the peak systolic velocity ratio on duplex ultrasound or greater than 50% arterial diameter narrowing based on angiography, and worsening symptoms in the target limb with at least a one-category decline in the Rutherford classification. The primary effective endpoint was defined as the 12-month primary patency rate and 18-month freedom rate from clinically driven target lesion revascularization (CD-TLR). All TLR cases were included as restenosis cases. The primary safety endpoint was defined as the 18-month rate of freedom from major adverse events (MAEs) comprising all-cause death, major (above-the-ankle) target limb amputation, or CD-TLR. Other endpoints comprised primarily sustained clinical improvement 18 months after the procedure. The primary sustained clinical improvement was defined as a sustained upward shift or at least one category in the Rutherford classification, with or without target lesion revascularization (TLR). Quality of life changes were evaluated using the Vascular Quality of Life Questionnaire (VascuQol-25) at the 18-month post-procedure timepoint.

### Statistical analysis

Continuous variables and outcomes are presented as the mean ± standard deviation. Categorical variables and outcomes are presented as absolute numbers and proportions of the study population. The cumulative primary patency at 12 months, along with freedom from the composite MAEs endpoint and CD-TLR rates over the 18-month follow-up period, were evaluated using the Kaplan–Meier method. All analyses were performed using SPSS 23.0 and Prism 8.0 software.

## Results

Between 2021 and 2022, a total of 124 limbs of 122 patients were treated with S.M.A.R.T. Flex stents at our clinical research sites. Among those patients, 105 patients (107 limbs) were included in this study, while 17 were excluded due to loss of contact after discharge. Patient and lesion characteristics are given in Table 1.

**Table 1:** Demographics, risk factors and lesion characteristics

Characteristics (*N* = 105 patients; 107 limbs)	Mean ± SD or *N* (%)
Age-years	72.0 ± 9.7
Male	76 (71.0%)
Diabetes mellitus	54 (50.9%)
Coronary artery disease	25 (23.4%)
Hypertension	88 (83.0%)
Hypercholesterolemia	40 (37.7%)
Atrial fibrillation	6 (5.7%)
COPD	1 (0.9%)
Cerebral infarction	17 (15.9%)
Current smoking	40 (37.4%)
Chronic renal failure	15 (14.0%)
Previous intervention	35 (32.7%)
Rutherford classification	
R 2	13 (12.2%)
R 3	36 (33.6%)
R 4	18 (16.8%)
R 5	36 (33.6%)
R 6	4 (3.7%)
TASC classification	
A	4 (3.7%)
B	26 (24.3%)
C	46 (43.0%)
D	31 (29.0%)
Lesion type	
De novo	92 (86.0%)
Restenosis	15 (14.0%)
Lesion length (cm)	24.9 ± 20.4
CTO (%)	76 (73.8%)
CTO length (cm)	20.3 ± 9.9
Calcification	
No/mild/moderate	69 (64.5%)
Severe	38 (35.5%)
Modified SVS run-off score	8.9 ± 5.2
Popliteal artery (P2–3) involvement	
Baseline ABI	0.4 ± 0.3
Baseline Vascqol score	2.8 ± 1.1

*COPD* chronic obstructive pulmonary disease, *TASC classification* The Trans-Atlantic Inter-Society Consensus classification, *SVS* Society for Vascular Surgery, *CTO* chronic total occlusion, *ABI* ankle brachial index

The mean age was 72 ± 9.7 years, and 71.0% of the study population was male. Most patients had hypertension (83.0%), and over half had diabetes (50.9%). The cohort included 33.6% with severe claudication (Rutherford category 3), 54.1% with chronic limb-threatening ischemia (CLTI), 16.8% with rest pain (Rutherford category 4), and 37.3% with tissue loss (Rutherford categories 5–6). 86.0% of lesions were treated for de novo lesions, and 14.0% of lesions were restenosis lesions. Seven patients had undergone prior stent implantation in target limbs before enrollment. 72.0% of lesions were TASC C and D lesions. The mean lesion length was 24.9 ± 20.4 cm, and the median and quartiles were 18.5 (10.5–26) cm. And 73.8% were chronic total occlusions (CTOs), the mean CTO lesion length was 20.3 ± 9.9 cm. 35.5% of lesions exhibited severe calcification. The mean modified SVS run-off score was 8.9 ± 5.2.

During the procedure, all the lesions were successfully crossed. In cases of CTOs, a retrograde approach was attempted in 23 of 107 limbs (21.5%) when the antegrade approach failed to re-enter the distal true lumen. Among the 107 limbs, 6 (5.6%) were identified with thrombosis in the target lesion, and mechanical thrombectomy was performed before stent implantation. In the present study, 50.5% of the limbs were treated with DCBs at the operator’s discretion, and 83.3% of TASC C/D lesions were primarily treated with DCBs. Additionally, 44.3% of the limbs underwent concomitant run-off revascularization. The mean modified SVS run-off score after the intervention was 6.0 ± 2.4. A total of 127 stents were placed in 107 limbs, with 1 stent placed in 88 limbs (82.2%), 2 stents placed in 18 limbs (16.8%), and 3 stents implanted in 1 limb (0.9%). The mean length of stented area was 21.8 ± 14.2 cm. In 6 cases (5.6%), the stent was deployed distal to the popliteal artery P3 segment (Table 2).

**Table 2:** Procedure and device characteristics

Variable (*N* = 107 limbs)	Mean ± SD or *N* (%)
Type of recanalization	
Antegrade recanalization	84 (78.5%)
Dual approach recanalization	23 (21.5%)
Atherectomy	
DCB used in target lesion	54 (50.5%)
Concomitant run-off recanalization	47 (44.3%)
Mean Stent length (cm)	21.8 ± 14.2
Number of stents per lesion	
1	88 (82.2%)
2	18 (16.8%)
3	1 (0.9%)
Stent diameter (mm)	
5	46 (42.99%)
6	58 (54.21%)
7	3 (2.80%)
Stent extending to distal popliteal artery	6 (5.6%)
Pre- procedural ABI	0.4 ± 0.3
Post- procedural ABI	0.8 ± 0.3
Pre-procedural modified SVS run-off score	8.9 ± 5.2
Post-procedural modified SVS run-off score	6.0 ± 2.4

*SD* standard deviation, *DCB* drug-coated balloon, *SVS* Society for Vascular Surgery, *ABI* ankle brachial index

The technical success rate was 95.3% (102/107). During the procedure, 5 patients (4.9%) experienced complications before their discharge. Two patients experienced local access site bleeding, one of whom required surgery, while the other was treated with manual compression. The third patient experienced perforation caused by the guide wire while crossing the lesion, which was successfully managed with intraluminal balloon dilation and manual compression applied outside the affected area. Two stenoses were induced at the access site by the procedure of using vessel closure devices, which were successfully treated with balloon dilation. After the procedure, the mean ABI score improved from a baseline of 0.4 ± 0.3 to 0.8 ± 0.3 (*P* < 0.01).

**Fig. 1:**
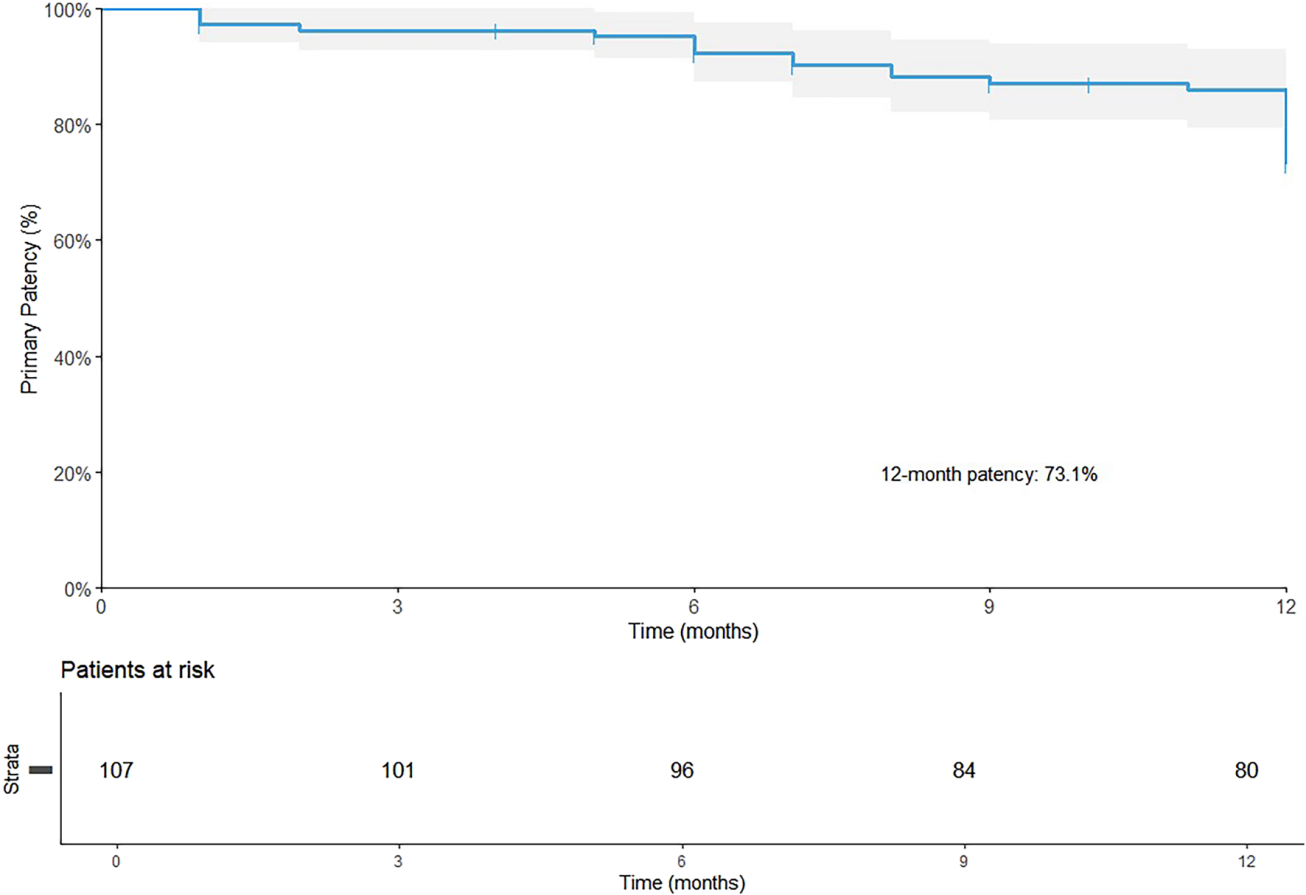
The 12-month primary patency rate

**Fig. 2:**
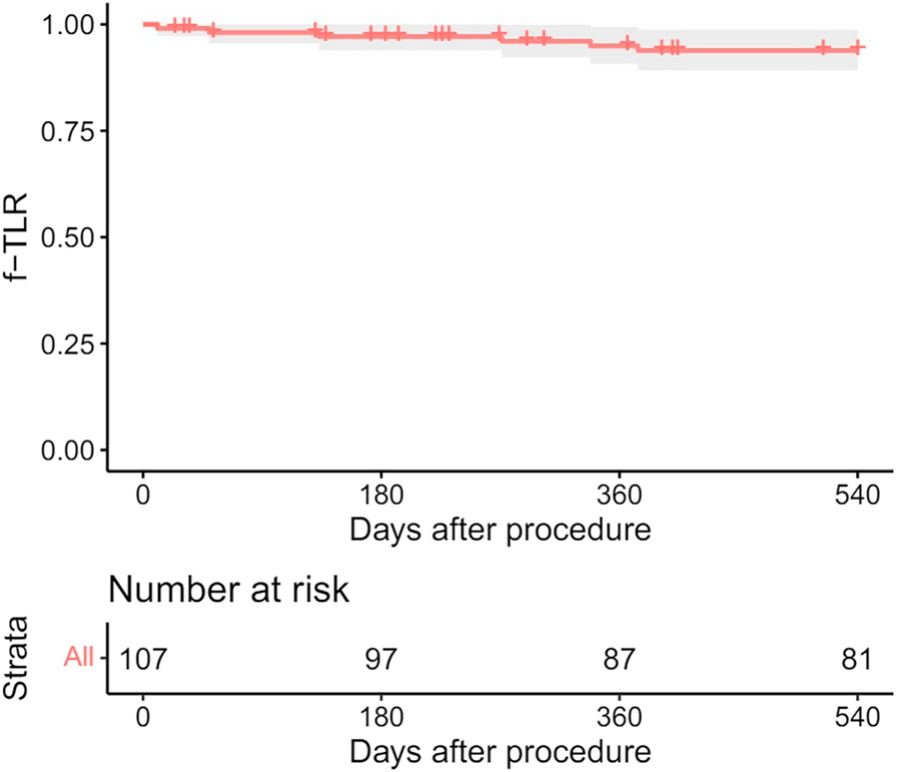
Freedom from CD-TLR 18 months after the procedure

The median follow-up period was 617 days (with an interquartile range of 417–783 days). Eleven patients (10.3%) were lost to follow-up. In addition, 41 (38.3%) patients underwent a duplex ultrasound examination at the 12-month follow-up. The 12-month primary patency rate was 73.1% (95% CI: 64.7–82.5%) (Fig. 1). The 18-month freedom from CD-TLR was 93.9% (95% CI: 89.2–98.8%) (Fig. 2).

**Fig. 3:**
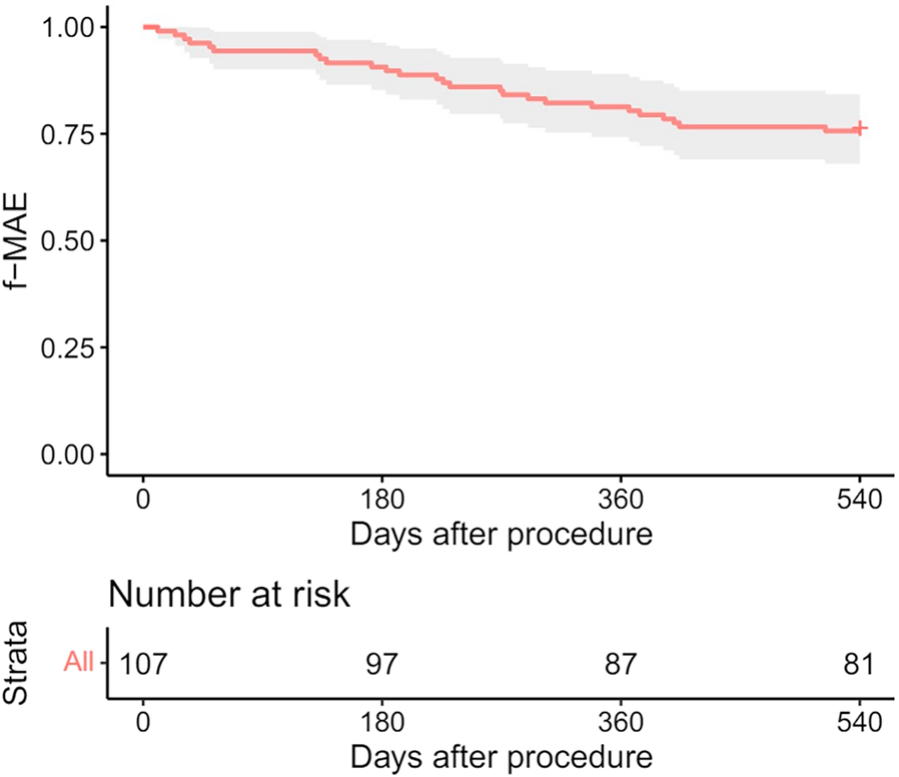
Freedom from MAEs through 18 months after the procedure

The primary safety endpoint, 18-month freedom from MAEs (all-cause death, major amputation, and target vessel revascularization), was 75.7% (95% CI: 68.0–84.3%) (Fig. 3).

**Fig. 4:**
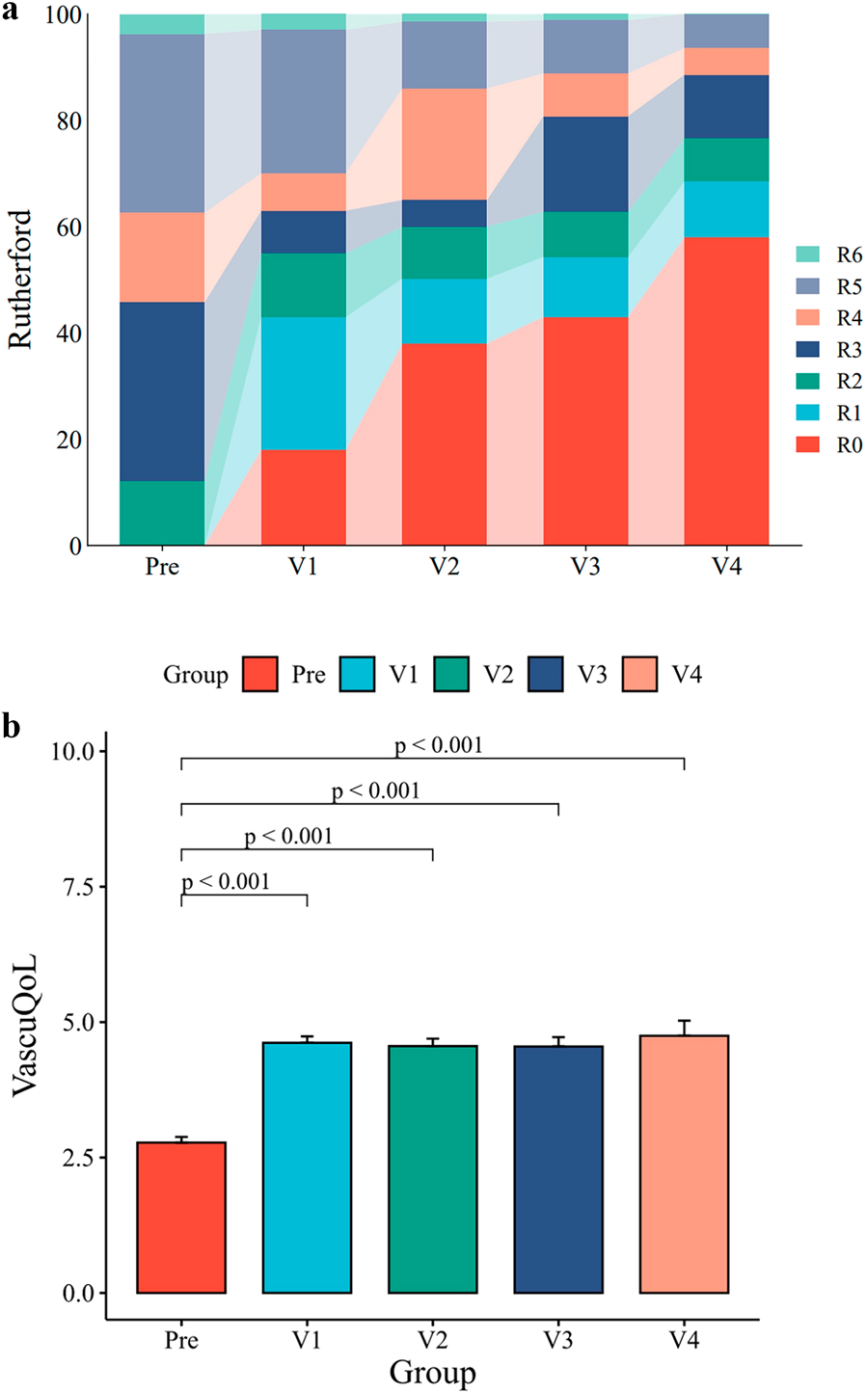
**A** Changes in the Rutherford category over 18 months. **B** Changes in the VascuQOL score over 18 months

The estimated freedom from major amputation in the target limb at 18 months was 95.0% (95% CI: 90.9–99.4%). The distribution of Rutherford clinical categories at baseline, 1, 3, 6, 12, and 18 months are shown in Fig. 4A.

Durable clinical improvement was achieved in 92.5% of patients at 18 months. VascuQOL total scores demonstrated significant improvement from baseline (2.8 ± 1.1) to 18-month follow-up (4.7 ± 1.4; *P* < 0.001), as shown in Fig. 4B.

Subgroup analyses between the DCB and non-DCB treatments (Table 3) for freedom from CD-TLR did not reveal any statistically significant differences (95.43 vs. 95.0%; *P* = 0.9).

**Table 3:** Lesion characteristics of the two groups

	Without DCB treatment (*N* = 53)	With DCB treatment (*N* = 54)	*P*
Rutherford classification			0.028
R 2	5 (9.43%)	8 (14.81%)	
R 3	26 (49.06%)	10 (18.52%)	
R 4	8 (15.09%)	10 (18.52%)	
R 5	13 (24.53%)	23 (42.59%)	
R 6	1 (1.89%)	3 (5.56%)	
TASC classification			0.083
A	3 (5.67%)	1 (1.85%)	
B	17 (32.08%)	9 (16.67%)	
C	18 (33.96%)	28 (51.85%)	
D	15 (28.30%)	16 (29.63%)	
CTO (%)	37 (69.81%)	42 (77.78%)	0.142
Severe calcification	16 (30.19%)	22 (40.74%)	0.279
Modified SVS run-off score	9.14 ± 5.17	8.59 ± 5.32	0.592

*DCB* drug-coated balloon, *TASC classification* The Trans-Atlantic Inter-Society classification, *CTO* chronic total occlusion

During the 18-month follow-up, 17 patients (16.2%) died, and 5 patients (4.8%) underwent major amputations. Seven patients died due to heart failure, 1 due to septic shock caused by moist gangrene in the target limb, 1 due to cerebral hemorrhage, 3 due to coronavirus infection, 1 due to a traffic accident, and 4 due to unclear reasons.

## Discussion

The present study demonstrated the effectiveness and safety of the S.M.A.R.T. Flex stent for treating FP occlusive lesions, including long and complex FP lesions with a mean lesion length of 25 cm and a 74% CTO rate. A real-world population of patients was included in this study, with a high proportion of patients with CLTI (54.1%). The acceptable low reintervention rate was 7.5% at 18 months, a primary sustained clinical improvement was observed in 92.5% of patients at 18 months, and the mean post-interventional score of quality of life was improved significantly at 18 months compared with pre-intervention.

Long and complex FP lesions pose a challenge due to poor patency rates caused by neointimal hyperplasia and smooth muscle cells proliferation. Several anti-restenosis technologies have been investigated to inhibit neointimal hyperplasia. One is the covered stent graft, which is effective in treating longer lesions by creating a mechanical barrier that prevents the in-growth of neointimal tissue [9–11]. Studies have shown a satisfactory primary patency rate of approximately 75% at 12 months and 79.4% freedom from TLR at 24 months [9–11]. Nevertheless, adherence to rigorous patient selection criteria is essential to mitigate acute thrombosis risk associated with these graft devices, thus restricting their application in complex lesions.

On the other hand, the delivery of anti-proliferative agents, such as paclitaxel, via stents or balloons has demonstrated efficacy in reducing restenosis in FP lesions [12]. Drug-eluting stent (DES) has shown superior primary patency compared to bare metal stents (BMS) or POBA [13, 14]. Still, to treat the whole lesion with an anti-proliferative drug, the lesion must be covered completely with the DES, which might be costly, especially for long lesions. Furthermore, long stenting increases the risk of stent fracture or restenosis [15]. In contrast to DES, DCB can treat all locations of the FP artery without impacting future treatment options by changing the anatomical structure or leaving stents behind [16, 17]. Schmidt et al. [4] demonstrated the effectiveness of DCB treatment even for long FP lesions, with a mean lesion length of 240 mm and a high proportion of chronic total occlusions (65.3%). Nevertheless, there was a greater risk of flow-limiting dissection and early vessel recoil, leading to stenting rates of 2.5–12% in short lesions and 23–45% in long lesions. Therefore, bare metal stents continue to play an important role in FP interventions.

Advances in stent technology have led to the development of new nitinol stents for treating complex and long lesions in the FP artery. The S.M.A.R.T. Flex stents feature a unique design with fully interconnected helical strut bands and flexible bridges, providing high radial force and axial compliance to prevent recoil and stent fracture [18]. Studies have evaluated the safety and efficacy of using S.M.A.R.T. Flex for treating FP lesions, but the published experience of S.M.A.R.T. Flex use has been limited to short and intermediate lesions [19, 20]. Indeed, the OPEN study included 257 subjects with a mean lesion length of 7.1 cm and reported a 12-month primary patency rate of 68.4%, with freedom from TLR rates of 84.7% at 12 months, 74.6% at 24 months, and 72.8% at 36 months. This retrospective study analyzed 80 patients receiving S.M.A.R.T. Flex stents for peripheral artery disease, with a mean lesion length of 80 mm. Kaplan–Meier estimates demonstrated 80% primary patency at 21 months [19].

The present study included patients with longer lesions and infrapopliteal involvement, yet similar effectiveness outcomes were still achieved compared with those of the above studies. In the present registry study, the S.M.A.R.T. Flex stents were used for femoropopliteal occlusive disease, including complex lesions with 25 cm mean length, 74% chronic total occlusions (CTOs), and 54.2% chronic limb-threatening ischemia (CLTI). The primary sustained clinical improvement rate was 92.5% at 18 months. These results may be attributed to the fact that 50.5% of the subjects underwent primary DCB treatment, especially for TASC II C and D FP lesions where DCB therapy demonstrated established primary patency benefits. When encountering complex lesions (TASC C/D lesions), the operators prefer DCB treatment. This may account for the absence of statistically significant differences between the two groups. Nevertheless, in the OPEN study [20], patients with lesions >18 cm were excluded, 52.5% had severe claudication, the mean lesion length was 7.1 ± 4.6 cm, and the 12-month patency rate was 68.4%. In the retrospective study by Garriboli et al. [19], the mean lesion length was 8.2 cm, and 7.5% of the patients experienced stent restenosis at 12 months. These findings indicate that the S.M.A.R.T. Flex stents are a safe and effective treatment option for long and complex FP lesions. Such results are also supported by observations made with other stent systems. The Innospring stent demonstrated 12-month and 24-month patency rates of 93.3% and 84.6%, respectively, when treating FP lesions with a mean length of 6.1 ± 3.5 cm [21]. The early results of the Supera stent yielded similar results [22], including complex FP lesions [23].

A real-world study of the Supera Peripheral Stent System to treat lesions of 14.3 ± 7.8 cm achieved patency rates of 72.6 and 60.8% at 12 and 24 months respectively [24]. These outcomes align with those of other real-world studies [25, 26]. In the STELLA SUPERA trial, FP lesions of 27.3 ± 12.7 cm were treated with the Supera stent, achieving clinical improvement rates of 87.2 and 79.7% at 12 and 24 months, respectively [27]. Similar outcomes were observed in the STELLA-SUPERA-SIBERIA registry study (mean length 20.5 ± 7.2 cm), with primary patency rates of 80.2 and 63.6% at 12 and 24 months [28]. Therefore, current literatures support the use of stenting for the management of long and complex FP lesions. Nevertheless, formal clinical trials are necessary to draw definitive conclusions regarding the use of the S.M.A.R.T. Flex stents in such lesions. Notably, although the S.M.A.R.T. Flex stents have demonstrated promising results, there are no head-to-head comparisons with other stents, and the comparative effectiveness remains unclear, underscoring the need for randomized clinical trials to address this question.

In the MAJESTIC trial evaluating the Eluvia paclitaxel-eluting stent for femoropopliteal lesions, the 12-month primary patency rate reached 96.1% [29], and the freedom from TLR rate was 85.3% at 3 years [30]. While demonstrating superior long-term outcomes compared to our cohort, the MAJISTIC population had significantly less complex disease, with a mean lesion length of 70.8 ± 28.1 mm. In our study, it was 249 ± 204 mm, and the prevalence of CTO and CLTI were 74 and 54.2%. In another Eluvia DES study with longer and more complex lesions (the mean lesion length was 194 ± 108 mm, 74% of the lesions were CTO), the Kaplan–Meier estimate of primary patency was 71% at 24 months, whereas both the secondary patency rate and freedom from TLR were 80% [31]. In this study, approximately 30% of patients had Rutherford categories greater than grade 3, whereas in our study, this group exceeded 50%.

The S.M.A.R.T. Flex stents demonstrate an acceptable safety profile, consistent with previous reports of low fracture incidence. In the OPEN study, the 24-month stent fracture rate was 4.3% with no additional fractures at 36 months [20]. A single-center analysis observed no fractures during the entire follow-up period [19]. In our cohort, 18-month all-cause mortality was 16.2% (including non-cardiovascular deaths) with a major amputation rate of 4.8%. Considering that the study period encompassed the COVID-19 pandemic, the higher mortality could be explained by the actual cases of death due to COVID-19, and by delayed healthcare-seeking behavior despite the presence of symptoms.

### Limitations

This real-world registry has inherent limitations. Even though 11 centers participated in the TALENT registry, the number of patients was small, owing to the relatively low rate of patients treated with the S.M.A.R.T. Flex stents. Predefined data collection variables constrained comprehensive analysis of factors influencing stent patency and complications.

Regarding clinical endpoints, the rate of angiographic follow-up was lower, and the freedom from CD-TLR may not accurately reflect the true progression of the lesions due to potential treatment selection bias, which may lead to under-detection of asymptomatic restenosis or disease progression. This limitation could contribute to both treatment selection bias and detection bias.

## Conclusion

Considering the objective performance of the S.M.A.R.T. Flex stent in this study, the stent demonstrated favorable efficacy and safety profiles in treating complex long-segment femoropopliteal lesions, including TASC II C/D lesions. These findings support its clinical utility for endovascular revascularization.

## Data Availability

No datasets were generated or analysed during the current study.

## References

[1] Parwani D, Ahmed MA, Mahawar A, et al. Peripheral arterial disease: a narrative review. Cureus. 2023;15(6): e40267. 37448414 10.7759/cureus.40267 PMC10336185

[2] Thukkani AK, Kinlay S. Endovascular intervention for peripheral artery disease. Circ Res. 2015;116(9):1599–613. 25908731 10.1161/CIRCRESAHA.116.303503 PMC4504240

[3] Li M, Tu H, Yan Y, et al. Meta-analysis of outcomes from drug-eluting stent implantation in femoropopliteal arteries. PLoS ONE. 2023;18(9): e0291466. 37733656 10.1371/journal.pone.0291466 PMC10513203

[4] Schmidt A, Piorkowski M, Görner H, et al. Drug-coated balloons for complex femoropopliteal lesions. JACC Cardiovasc Interv. 2016;9(7):715–24. 27056311 10.1016/j.jcin.2015.12.267

[5] Vu MH, Sande-Docor GM, Liu Y, et al. Endovascular treatment and outcomes for femoropopliteal in-stent restenosis: insights from the XLPAD registry. J Interv Cardiol. 2022;2022:5935039. 35911663 10.1155/2022/5935039 PMC9307356

[6] Qato K, Conway AM, Mondry L, et al. Management of isolated femoropopliteal in-stent restenosis. J Vasc Surg. 2018;68(3):807–10. 30144908 10.1016/j.jvs.2018.01.030

[7] Liu Y, Wang Q, Wu Z, et al. A prospective, multicenter, real-world observational study evaluating the impact of tibial runoff on clinical outcomes after endovascular therapy for femoropopliteal lesions: research protocol. Front Cardiovasc Med. 2022;9:1035659. 36465469 10.3389/fcvm.2022.1035659 PMC9709143

[8] von Elm E, Altman DG, Egger M, et al. The Strengthening the Reporting of Observational Studies in Epidemiology (STROBE) statement: guidelines for reporting observational studies. Ann Intern Med. 2007;147:573–7. 17938396 10.7326/0003-4819-147-8-200710160-00010

[9] Sarradon P, Ozdemir BA, Becquemin JP. Technique and early results of percutaneous femoropopliteal bypass with stent graft. J Vasc Surg Cases Innov Tech. 2023;9(4): 101317. 37841528 10.1016/j.jvscit.2023.101317 PMC10569994

[10] van Nistelrooij AMJ, van’t Sant HP, Schouten O. Covered stent grafts for relining of chronically occluded femoro-popliteal bypasses in frail patients. Clin Case Rep. 2021;9(8):e04647. 34430011 10.1002/ccr3.4647 PMC8365545

[11] Hajibandeh S, Hajibandeh S, Antoniou SA, et al. Covered vs uncovered stents for aortoiliac and femoropopliteal arterial disease: a systematic review and meta-analysis. J Endovasc Ther. 2016;23(3):442–52. 27099281 10.1177/1526602816643834

[12] Zhang C, Yin G. Safety of paclitaxel-coated devices in the femoropopliteal arteries: a systematic review and meta-analysis. PLoS ONE. 2022;17(10): e0275888. 36227807 10.1371/journal.pone.0275888 PMC9560511

[13] Meraj PM, Jauhar R, Singh A. Bare metal stents versus drug eluting stents: where do we stand in 2015? Curr Treat Options Cardiovasc Med. 2015;17(8):393. 26154294 10.1007/s11936-015-0393-y

[14] Varetto G, Gibello L, Boero M, et al. Angioplasty or bare metal stent versus drug-eluting endovascular treatment in femoropopliteal artery disease: a systematic review and meta-analysis. J Cardiovasc Surg (Torino). 2019;60(5):546–56. 31527577 10.23736/S0021-9509.19.11115-9

[15] Claessen BE, Henriques JP, Jaffer FA, et al. Stent thrombosis: a clinical perspective. JACC Cardiovasc Interv. 2014;7(10):1081–92. 25341705 10.1016/j.jcin.2014.05.016

[16] Keefe N, Shull T, Botea L, et al. Drug-coated balloon versus drug-eluting stent: the debate of leave nothing behind. Semin Intervent Radiol. 2023;40(2):161–6. 37333737 10.1055/s-0043-57261 PMC10275675

[17] Mohapatra A, Saadeddin Z, Bertges DJ, et al. Nationwide trends in drug-coated balloon and drug-eluting stent utilization in the femoropopliteal arteries. J Vasc Surg. 2020;71(2):560–6. 31405761 10.1016/j.jvs.2019.05.034 PMC7007839

[18] Maleckis K, Deegan P, Poulson W, et al. Comparison of femoropopliteal artery stents under axial and radial compression, axial tension, bending, and torsion deformations. J Mech Behav Biomed Mater. 2017;75:160–8. 28734257 10.1016/j.jmbbm.2017.07.017 PMC5623954

[19] Garriboli L, Miccoli T, Pruner G, et al. PTA and stenting of femoropopliteal trunk with cordis Smartflex stent system: a single-center experience. Vasc Endovascular Surg. 2020;54(1):17–24. 31526118 10.1177/1538574419875551

[20] Gray WA, Cardenas J, Teigen CL. Evaluation of safety and efficacy of the S.M.A.R.T.® Flex vascular stent system (OPEN study). Catheter Cardiovasc Interv. 2022;100(6):1078–87. 36177491 10.1002/ccd.30414

[21] Yang S, Ni Q, Wang W, et al. Novel self-expanding interwoven nitinol stent for treating femoropopliteal artery disease: 12-month results of single-center first-in-man study. J Endovasc Ther. 2023;31(5):955–63. 36927094 10.1177/15266028231159243

[22] Garcia L, Jaff MR, Metzger C, et al. Wire-interwoven nitinol stent outcome in the superficial femoral and proximal popliteal arteries: twelve-month results of the SUPERB Trial. Circ Cardiovasc Interv. 2015;8(5):e000937. 25969545 10.1161/CIRCINTERVENTIONS.113.000937

[23] Werner M, Paetzold A, Banning-Eichenseer U, et al. Treatment of complex atherosclerotic femoropopliteal artery disease with a self-expanding interwoven nitinol stent: midterm results from the Leipzig SUPERA 500 registry. EuroIntervention. 2014;10(7):861–8. 24682531 10.4244/EIJV10I7A147

[24] Van Meirvenne E, Reyntjens P, Tielemans Y. Self-expanding interwoven nitinol stent in severe femoropopliteal arterial disease. Real life results of the Supera Peripheral Stent System®. Acta Chir Belg. 2023;123(5):463–72. 35485637 10.1080/00015458.2022.2072457

[25] Salamaga S, Stepak H, Zolynski M, et al. Three-year real-world outcomes of interwoven nitinol supera stent implantation in long and complex femoropopliteal lesions. J Clin Med. 2023;12(14):4869. 37510984 10.3390/jcm12144869 PMC10381725

[26] Myint M, Schouten O, Bourke V, et al. A real-world experience with the supera interwoven nitinol stent in femoropopliteal arteries: midterm patency results and failure analysis. J Endovasc Ther. 2016;23(3):433–41. 27004494 10.1177/1526602816639543

[27] Nasr B, Gouailler F, Marret O, et al. Treatment of long femoropopliteal lesions with self-expanding interwoven nitinol stent: 24 month outcomes of the STELLA-SUPERA trial. J Endovasc Ther. 2023;30(1):98–105. 35114841 10.1177/15266028221075227

[28] Gostev AA, Osipova OO, Cheban AV, et al. Treatment of long femoropopliteal occlusive lesions with self-expanding interwoven nitinol stent: 24 month outcomes of the STELLA-SUPERA-SIBERIA register trial. J Endovasc Ther. 2025;32(1):192–8. 37128865 10.1177/15266028231170125

[29] Müller-Hülsbeck S, Keirse K, Zeller T, et al. Twelve-month results from the MAJESTIC Trial of the eluvia paclitaxel-eluting stent for treatment of obstructive femoropopliteal disease. J Endovasc Ther. 2016;23(5):701–7. 27193308 10.1177/1526602816650206 PMC5023034

[30] Müller-Hülsbeck S, Keirse K, Zeller T, et al. Long-term results from the MAJESTIC Trial of the eluvia paclitaxel-eluting stent for femoropopliteal treatment: 3-year follow-up. Cardiovasc Intervent Radiol. 2017;40(12):1832–8. 28948322 10.1007/s00270-017-1771-5

[31] Stavroulakis K, Torsello G, Bosiers M, et al. 2-Year outcomes of the eluvia drug-eluting stent for the treatment of complex femoropopliteal lesions. JACC Cardiovasc Interv. 2021;14(6):692–701. 33736776 10.1016/j.jcin.2021.01.026

